# Improved lymphatic targeting: effect and mechanism of synthetic borneol on lymph node uptake of 7-ethyl-10-hydroxycamptothecin nanoliposomes following subcutaneous administration

**DOI:** 10.1080/10717544.2018.1482973

**Published:** 2018-06-14

**Authors:** Tiantian Ye, Yue Wu, Lei Shang, Xueqing Deng, Shujun Wang

**Affiliations:** a Department of Pharmaceutics, School of Pharmacy, Shenyang Pharmaceutical University, Shenyang, China;; b Department of Pharmaceutics, School of Chinese Medicines, Shenyang Pharmaceutical University, Shenyang, China;; c Shenyang Medical College, Shenyang, China

**Keywords:** Synthetic borneol, lymphatic targeting, lymph node uptake, 7-ethyl-10-hydroxycamptothecin, nanoliposome

## Abstract

Borneol as a penetration enhancer is widely used in guiding other components through the biological barrier into the targeting organs or tissues. This study aimed at studying effect and mechanism of synthetic borneol (S-BO) on improving lymphatic-targeting ability of 7-ethyl-10-hydroxycamptothecin liposomes (SN-38-Lips) via increasing lymph node uptake. At first, SN-38-Lips prepared had appropriate particle distribution, drug loading property and compatible stability with S-BO. Both *in vitro* cellular uptake and *in vivo* fluorescence imaging showed that 2 and 5 mg/mL S-BO, especially 2 mg/mL S-BO, enhanced cytoplasmic fluorescence signal of SN-38-Lips in the macrophages based on phagocytosis effect. And high-intensity zone appeared in the paracortex and medulla of popliteal lymph node. SN-38-Lips were subcutaneously (s.c.) injected into the right footpad of KM rats in the dose of 4 mg/kg following s.c. injection of 1, 2 and 5 mg/mL BO suspension. The lymphatic pharmacokinetics were investigated to explore the promotion law of S-BO, and combined with tissue irritation to optimize S-BO concentrations. The results indicated that 2 mg/mL S-BO could reduce injection-site retention, and prolong residence time and increase uptake of lymph nodes, which would not cause inflammatory reaction of injection site. In conclusion, the present study may provide a basic study for improving lymphatic-targeting ability of SN-38-Lips by the S-BO regulation, and to be the helpful guidance for further study in lymphatic targeting of delivery system.

## 1. Introduction

The lymphatic system as an additional circulatory system, consisting of lymphoid organ, lymph and lymphatic pathways, plays an active role in recognition and response of the immune system to disease (Swartz, 2001). And most solid cancers initially metastasize into the lymph nodes before hematological dissemination (Mcallaster & Cohen, 2011). Hence, the lymphatic system has become an important target and drug delivery route for preferential therapeutic and diagnostic agent and improving bioavailability of poorly soluble and unstable drugs that undergo hepatic first-pass metabolism (Singh et al., 2014). The multidisciplinary researchers pay close attention to the improvement of lymphatic targeting (Yang et al., 2015). The lymphatic targeting contains both two processes of lymphatic drainage evaluated by lymphatic penetration and lymphatic retention evaluated by lymph node uptake. The common methods of improving the lymphatic targeting (Moghimi & Moghimi, 2008; Willis et al., 2012) are to adjust interstitial osmotic pressure for increasing lymphatic drainage (Yang et al., 2015) or to increase molecular weight (Cohen et al., 2009), loaded in the drug delivery system for enhancing lymphatic uptake.

Borneol (BO) is a monoterpenoid component, being classified into nature borneol and synthetical borneol (S-BO). S-BO is produced by turpentine through a series of synthesis processes, containing d-borneol and isoborneol. S-BO is widely used in medicine because they are easily available, inexpensive and have same pharmacodynamics as nature borneol (Yu et al., 2007). According to the basic theories of traditional Chinese medicine, borneol is called a ‘penetration enhancer’, which not only can lead other components into the brain through blood–brain barrier (Tao et al., 2017; Yin et al., 2017), but also enhance other tissues targeting (Zhang et al., 2015) via improving the permeability of various physiological barrier such as the skin (Mai et al., 2003; Yi et al., 2016), mucous membranes (Lu et al., 2012; Chen et al., 2014) and gastrointestinal tract (Zhou et al., 2010; Ru et al., 2016). However, there is no relevant previous study to investigate the impact of S-BO on lymphatic targeting of drug delivery system by evaluating lymph node uptake.

7-Ethyl-10-hydroxycamptothecin (SN-38) is an active form metabolized from irinotecan hydrochloride (CPT-11) as a prodrug against several tumor cell lines. SN-38 may be directly applied to cancer treatment over CPT-11 as a potentially highly effective anticancer agent. Nonetheless, its major problems are the insolubility in water as well as physiologically acceptable organic solvents and its severe toxicity, which limited it use in clinic. Liposomes can improve chemical stability increasing drug solubility and reducing toxicity of free anticancer drugs. And liposome is one of the well-studied nanocarrier to deliver the therapeutic and diagnostic agents to enhance lymphatic exposure (Cai et al., 2011). Thus, liposomes delivery system is selected to carry SN-38 to increase the solubility, improve *in vitro* and *in vivo* stability, reduce adverse reactions and enhance the lymphatic exposure. In this study, we selected SN-38 nanoliposomes (SN-38-Lips) as a model drug delivery system to study the effect of S-BO on lymphatic targeting.

In this study, we aimed at evaluating the overall impact of S-BO on lymphatic targeting of SN-38-Lips via *in vivo* fluorescence imaging and lymphatic pharmacokinetics. In addition, *in vitro* cell uptake and *in vivo* intra-lymph node distribution were investigated to explore lymphatic target mechanism. At last, the irritation of subcutaneous injection site and lymph node of synthetic borneol was observed by Hematoxylin and Eosin (H&E) staining.

## 2. Materials and methods

### 2.1. Materials and animals

The S-BO was provided by Kaoji Yufeng Natural Plant Spice Co., Ltd., (Jinan, China). The molecular formula of BO is C_10_H_18_O and the molecular weight is 154.25. SN-38, the purity of which was over 99%, was purchased from Jingmen Shuaibang Chemical Science and Technology Co. Ltd., Jingmen, Hubei, China. The molecular formula and molecular weight of SN-38 are C_22_H_20_N_2_O_5_ and 392.4, respectively. Soybean phospholipid (SPC, LIPOID S100) was obtained from Germany Lipoid GmbH Co. Ltd., Ludwigshafen, Germany. Phosphatidyl ethanolamine (PE) was supplied by Shanghai Advanced Vehicle Technology Ltd Co, Shanghai, China. Cholesterol (Chol) was supplied by Tianjin Bodi Chemical Holding Co., Ltd., Tianjin, China. All chemicals and reagents used were analytical grade or better.

Kunming species male mice (KM mice) (18–22 g) were provided from the Laboratory Animal Center, Shenyang Pharmaceutical University (Shenyang, China). All the experimental protocols adhered to the principles of laboratory animal care and were approved by the Experimental Animal Use and Care Committee, Shenyang Pharmaceutical University.

### 2.2. Formulation preparation

#### 2.2.1. Preparation of S-BO suspension

The S-BO were ground and then sieved by the 150 mesh sieve. The sieved BO powders were added into and well mixed with glycerin (3:1, w:w), following by diluting with water. The S-BO suspensions were sonicated at 400 W for 10 min using probe sonicator (S-4000-010; Misonix Inc., Farmingdale, NY).

#### 2.2.2. Preparation of SN-38-Lips

According to our previous study (Wang et al., 2013), SN-38-loaded nanolipsomes (SN-38-Lips) were prepared by thin-film hydration and sonication method. The formulation of SN-38-Lips prepared for lymphatic target study as follows: SPC/PE/Chol/SN-38 = 30:5:16:4 (molar ratio). Briefly, a mixture of SPC/PE/Chol/SN-38 were dissolved in chloroform and dried into a thin film by a rotary evaporator (RE52CS; Shanghai Yarong Bio-Chem Instruments, Shanghai, China). The film was desiccated under vacuum overnight, and hydrated with distilled water at 50 °C water baths. The resulting solution was sonicated at 400 W for 5 min, and then filtered through the 0.8 μm filter membrane.

### 2.3. *In vitro* characterization of SN-38-Lips

The morphologies of SN-38-Lips were investigated using transmission electron microscopy (TEM) (JEM-1200EX; JEOL Inc., Tokyo, Japan). The particle size distribution and zeta potential of SN-38-Lips were measured by laser light scattering using a Zetasizer Nano-ZS90 (Malvern Instruments Ltd., Malvern, UK) at 25 °C. The entrapment efficiency (EE) of SN-38-Lips was assayed by mini-column centrifugation method. In order to investigate the impact of S-BO on stability of SN-38-Lips, S-BO suspension with 1, 2 and 5 mg/mL of concentrations were incubated with SN-38-Lips (1:2, v:v) at 37 °C, and then EE, particle size and zeta potential of SN-38-Lips were measured at 0.5, 1, 2, 4, 8 and 12 h during incubation.

### 2.4. Cellular uptake study of macrophages

The impact of S-BO on the macrophages phagocytosis of SN-38-Lips was studied by fluorescence imaging of RAW264.7 cell lines. The cellular uptake was evaluated by a confocal laser scanning microscopy (CLSM) (TCS SP5 II; Leica Microsystems CMS GmbH, Mannheim, Germany). SN-38 solution was prepared by SN-38 dissolved in 400 μL, pH 2.6 sodium acetate–acetic acid buffer solution containing 0.03% (w:v) Tween-80. The RAW264.7 cells were incubated with SN-38 solution, alone SN-38-Lips group and SN-38-Lips combined with S-BO suspension groups in culture medium for 2 h. After that, the cells were washed twice with cold PBS and fixed with 4% paraformaldehyde solution for 10 min. Then, the cells were washed twice with cold PBS. The nuclei were stained by DAPI for another 30 min. The fixed cell monolayer was finally washed twice with PBS. The cover slips were placed onto the glass microscope slides and visualized by CLSM.

### 2.5. Fluorescence imaging study of lymph node

SN-38-Lips (the dose of 4 mg/kg) combined with 10 μL S-BO suspension with the concentrations of 1, 2 and 5 mg/mL were subcutaneously (s.c.) injected into the right rear footpad of KM rats (*n* = 5). SN-38-Lips were used as control. At 30 min, 1 h, 6 h post-injection, the popliteal lymph nodes (PLNs) were removed. The pseudo-color fluorescence images of PLNs were detected by the *in vivo* imaging system (Carestream Image Station System FX Pro, Carestream Health, Inc., Rochester, NY). At 1 h post-injection, the 5 μm frozen sections of isolated PLNs were prepared. Collect specimens on clean poly-l-lysine-coated glass slides and dry at room temperature overnight. Fix sections in acetone at 4 °C for 15 min, then thoroughly air-dry at room temperature. Rhodamine-phalloidin solution incubated the sections at 37 °C for 90 min. Then, 90% glycerol with DAPI covered the sections for fluorescence observation. The fluorescence imaging of sections was observed by CLSM.

### 2.6. Lymphatic pharmacokinetic study

About 10 μL of S-BO with the concentration of 1, 2 and 5 mg/mL were injected s.c. to the right rear footpad of KM rats (*n* = 5). Then, SN-38-Lips were injected s.c. at the dose of 4 mg/kg at 3 min post-injection. At the time point of 5 min, 10 min, 30 min, 1 h, 2 h, 4 h, 6 h, 12 h, and 24 h post s.c. administration of S-BO, blood was collected by extracting eyeballs and then lymph nodes and hind limb soles were harvested immediately, respectively. Samples were thawed to room temperature before dealing with them. Then, the tissues were homogenized with saline (foot homogenized with saline at the ratio of 1:10, g/mL) to get tissue homogenate samples. Exactly, 50 µL of tissue homogenate sample or plasma was transferred to a 1.5 mL EP together with 200 µL acetonitrile (containing 0.5% acetic acid, v/v), following by centrifuging at 15,435*g* for 10 min after vigorously vortexed for 3 min. The supernatant was transferred to another 1.5 mL EP. About 200 µL supernatant was used for fluorescent analyses. Fluorescent analyses of SN-38 *in vivo* experiments (200 µL per well) were carried out using a FLU-HPLC system, composed of a LC-10AT quaternary pump (Shimadzu Corporation, Shimadzu, Japan), a SIL-10AF autosampler (Shimadzu Corporation), a RF-20 fluorescence detector (Ex228 nm, Em543 nm, Shimadzu Corporation). Century SIL C18-BDS column (200 × 4.6 mm, 5 μm) (Bischoff GmbH, Muggensturm, Germany) was used at 25 °C. The mobile phase was 25 mM NaH_2_PO_4_ (pH =3.1) buffer:acetonitrile (67:33, v:v) and the flow rate was 1.0 mL/min. The injection volume was 10 μL. Non-compartmental pharmacokinetics (PK) parameters were carried out by DAS 2.0 software (Mathematical Pharmacology Professional Committee of China, Shanghai, China) by the statistical moment method.

### 2.7. Injection site and lymph node irritation

Effect of S-BO on the irritation of injection site and morphology of lymph node was histopathologically investigated using H&E staining. Exactly, 10 μL of S-BO with the concentration of 1, 2 and 5 mg/mL were injected s.c. to the right rear footpad of KM mice (*n* = 5). Then, at Day 3 post-injection, the claw pads were surgically removed and were fixed in 10% formalin, embedded in paraffin blocks, and sectioned at 4–5 μm cuts for H&E staining.

### 2.8. Statistical analysis

All the experimental date was subjected to statistical analysis, a value of *p* < .05 was regarded as significant. Data are expressed as mean and standard deviation and all the statistics analyses were performed by using SPSS17.0 (SPSS Inc., Chicago, IL). The tumor growth curves were analyzed by Mann–Whitney U-test.

## 3. Results and discussion

### 3.1. *In vitro* characterization of SN-38-Lips

**Figure 1.**
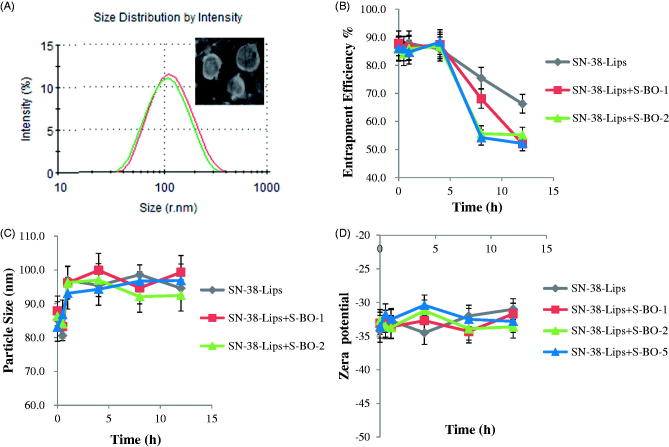
(A) Particle size distribution and morphology of SN-38-Lips and the effect of S-BO with the concentration of 1, 2 and 5 mg/mL on (B) entrapment efficiency %, (C) size distribution and (D) zeta potential of SN-38-Lips (mean ± SD, *n* = 3).

SN-38-Lips were the milky white suspensions with slightly blue opalescence. The shape of liposome was circular appeared in Figure 1(A). The average particle diameter of SN-38-Lips was 82.30 ± 1.46 nm measured by the DLS and PDI was 0.4 that mean particle size distribution was relatively narrow (Figure 1(A)). Particle size of nanoliposome was the key factor for lymphatic uptake (Oussoren et al., 1997). For small liposomes (<0.1 μm), the degree of lymphatic drainage could reach levels up to 70% of the injected dose (Allen et al., 1993). The EE of SN-38-Lips was 85.8 ± 1.3%. SN-38-Lips had a low zeta potential value of −33.78 ± 1.25 mV based on PE included in the prescription. Stability of liposome suspensions is highly dependent on surface charge or zeta potential. Particles with an absolute zeta potential value >30 mV have relatively high repulsive interaction and are considered to be stable. The results showed that SN-38-Lips as a model drug delivery system had suitable physicochemical properties for *in vivo* lymphatic uptake study.

Effects of different concentrations of S-BO on SN-38-Lips were evaluated by observing the changes of EE, particle size and zeta potential. The results in Figure 1 demonstrated that the EE of four formulations (Figure 1(B)) remained stable in 4 h. After 4 h post-incubation, the EE of SN-38-Lips gradually decreases in the four SN-38-Lips groups, while that decrease faster in the SN-38-Lips combined S-BO group. When the S-BO concentration was ≥2 mg/mL, the drug leakage of SN-38-Lips reached the maximum, which showed a fast decrease followed by a plateau from 8 h. This result implied that B-SO could decrease the EE of SN-38-Lips with time, which may due to the fact that S-BO could accelerate the movement of biofilm (Chen et al., 2014). The particle size (Figure 1(C)) and zeta potential (Figure 1(D)) of SN-38-Lips didn’t showed apparent change when were incubated with S-BO suspension during 12 h. It indicated that co-administration of S-BO suspension and SN-38-Lips don’t lead to changes in liposome morphology. In order to avoid the S-BO accelerate drug leakage, S-BO suspensions and SN-38-Lips are not mixed prior to administration and are administered sequentially.

### 3.2. Cellular uptake of SN-38-Lips combined with S-BO

**Figure 2.**
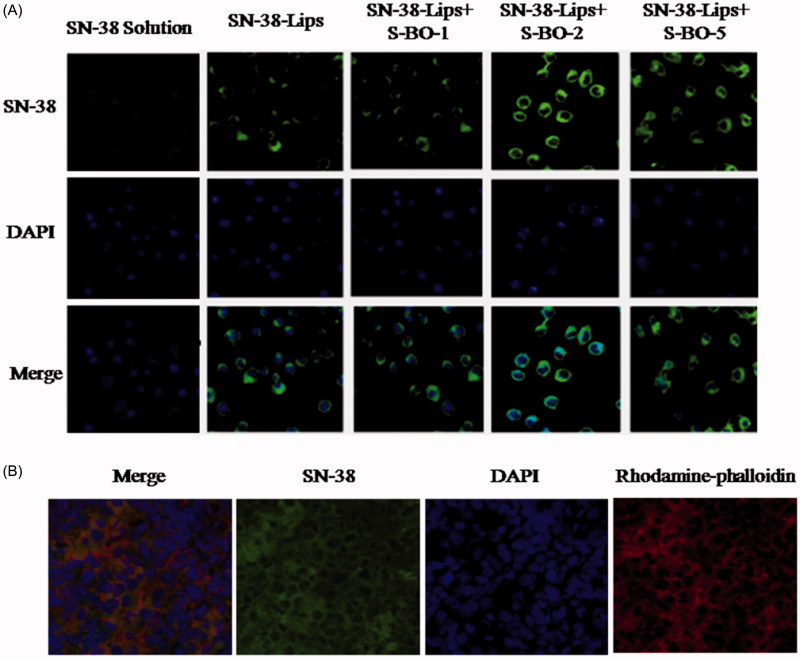
(A) *In vitro* cellular uptake of RAW264.7 cell line treated with SN-38 solution, SN-38-Lips and SN-38-Lips combined with S-BO in the concentrations of 1, 2 and 5 mg/mL. (B) Fluorescent image at ×400K magnification in the medulla of PLNs at 1 h post-administration via s.c. injection of SN-38-Lips combined with 2 mg/mL S-BO.

The results of the cellular uptake behavior could be observed on the merged channel of SN-38 in Figure 2. As presented in Figure 2(A), fluorescent signals of RAW264.7 cells treated with various SN-38-Lips formulation were obviously visualized in the cytoplasm. And the intracellular fluorescent intensity of SN-38-Lips formulations was more than that of SN-38 solution. This phenomenon could be explained by the fact that liposomes are the most widely studied carrier in deliver drugs targeting to macrophages by phagocytosis, but only a small fraction of soluble drug could reach the macrophages (Ahsan et al., 2002). In the liposome groups, the SN-38 fluorescent intensity of RAW274.7 cells incubated with combination of SN-38-Lips and S-BO suspension with 2 and 5 mg/mL concentration was more than alone SN-38-Lips. Thus the intracellular fluorescent intensity was in descending order of SN-38-Lips in combination of 2 mg/mL S-BO group, SN-38-Lips in combination of 5 mg/mL S-BO group, alone SN-38-Lips group and SN-38-Lips in combination of 1 mg/mL S-BO group. About 2 mg/mL S-BO could significantly increase the fluorescent intensity of SN-38-Lips in the RAW264.7 cells. This phenomenon could be explained by previous study. It had been reported that nature borneol significantly enhanced the cellular uptake of amino acids (Su et al., 2013). *In vivo* cellular uptake of SN-38-Lips combined with 2 mg/mL S-BO in the paracortex zone of PLNs was showed in Figure 2(B). The fluorescent signal of DAPI, SN-38 and rhodamine-phalloidin indicated that both lymphocyte and macrophage in the paracortex zone of PLNs have cell nucleus appearing round or oval, but the macrophages have a broader cytoplasm. SN-38-Lips distributed in the cytoplasm of lymphocyte and macrophages, and there was higher fluorescence intensity in the macrophage cytoplasm. This result implied S-BO played a role on improving lymphatic uptake of SN-38-Lips via increase cellular uptake of lymphocyte and macrophages in the lymph node, especially through phagocytosis of macrophages.

### 3.3. *In vivo* distribution of SN-38-Lips combined with S-BO into the lymph node

**Figure 3.**
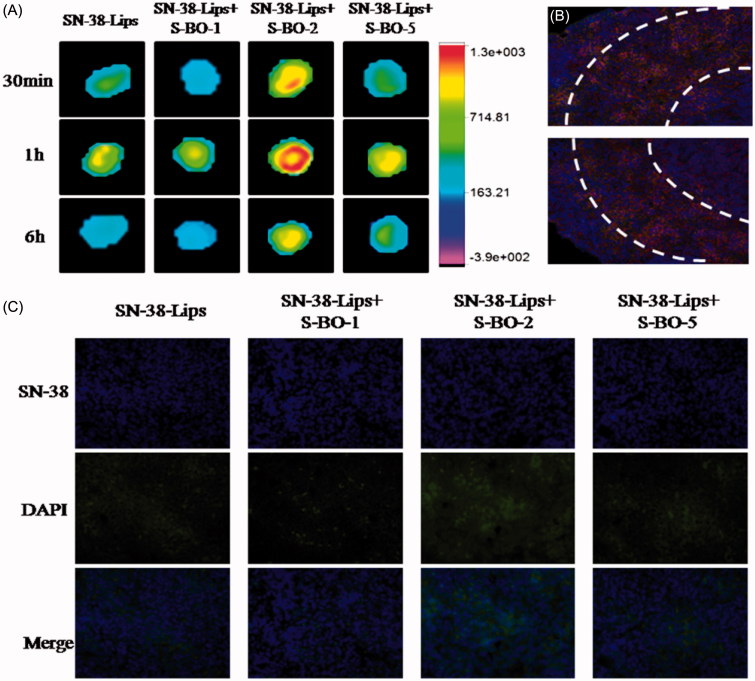
*In vivo* fluorescence imaging of PLNs subcutaneously injected with SN-38-Lips and SN-38-Lips combined with S-BO in the concentrations of 1, 2 and 5 mg/mL at 30 min, 1 h and 6 h. (B) Fluorescent image at ×200K magnification in the medulla of PLNs at 1 h post-administration via s.c. injection of SN-38-Lips combined with 2 mg/mL S-BO. (C) Fluorescent image at ×400K magnification in the medulla of PLNs at 6 h post-administration via s.c. injection of SN-38-Lips and SN-38-Lips combined with 1, 2 and 5 mg/mL S-BO.

*In vivo* fluorescent mapping ability of SN-38-Lips formulations was compared by NIR images and fluorescent intension. NIR fluorescence image was photographed at 30 min, 1 h and 6 h post-injection. As shown in Figure 3(A), near-infrared fluorescence imaging obviously could be observed in the paracortex zone and medulla of PLNs, indicating that SN-38-Lips successfully transferred to PLNs after the subcutaneous injection. Comparing with SN-38-Lips group, SN-38-Lips combined with 2 and 5 mg/mL S-BO group had higher NIR fluorescence intensity and better imaging effect. Moreover, SN-38-Lips combined with 2 mg/mL S-BO group reached the highest fluorescence signal at 1 h post-injection and fluorescent signals showed a circular distribution in the paracortex zone of lymph nodes. In the SN-38-Lips combined with 2 mg/mL S-BO group, this circular fluorescence distribution of the paracortex zone was also observed in the cryo-sections of PLNs at 1 h post-injection (Figure 3(B)). The fluorescence intensity of different SN-38-Lips formulation also was compared in the cryo-sections of PLNs at 6 h post-injection (Figure 3(C)). The SN-38 fluorescent intensity of the medulla zone of PLNs in the combination of SN-38-Lips and S-BO suspension (2 and 5 mg/mL of concentration) group was more than alone SN-38-Lips group. Thus the fluorescent intensity of SN-38 uptake in the lymph nodes was in descending order of SN-38-Lips in combination of 2 mg/mL S-BO group, SN-38-Lips in combination of 5 mg/mL S-BO group, alone SN-38-Lips group and SN-38-Lips in combination of 1 mg/mL S-BO group. The results were consistent with NIR fluorescence images of PLNs at 6 h post-injection.

### 3.4. Method validation tests

In the pharmacokinetic experiment, the method of FLU-HPLC and camptothecin as the internal standard were used. The chromatograms showed stable baselines and SN-38, camptothecin and endogenous substances in biological samples had been separated well. The limit of quantization (LOQ) was determined to be 7 ng/mL and the limit of detection (LOD) was 3 ng/mL in the present conditions. The regression equation of plasma sample was A = 0.000186C + 0.013650 (*r* = 0.9950) in the range of 7–500 ng/mL. The regression equation of foot sample was A = 1.5561X − 0.0315 (*r* = 0.9964) in the range of 0.5–50 μg/mL. The regression equation of lymph node sample was A = 1.5561X − 0.0315 (*r* = 0.9926) in the range of 0.5–50 μg/mL. The extract recovery of SN-38 in rat plasma, foot and lymph node were 86.5, 80.1 and 83.7%. The relative standard deviation of intra- and inter-day precision were <8.5%.

### 3.5. *In vivo* lymphatic drainage and non-compartmental pharmacokinetic analysis

Concentration versus time curves and non-compartmental pharmacokinetic parameters of injection site and plasma obtained from mice s.c. injecting SN-38-Lips group and SN-38-Lips group combined with S-BO in the concentrations of 1, 2 and 5 mg/mL are shown in Figure 4(A,B) and Table 1.

Figure 4.SN-38 concentration–time curve of (A) foot, (B) plasma, (C) PLNs, (D) ILNs and (E) RLNs in the SN-38-Lips group and SN-38-Lips group combined with S-BO in the concentrations of 1, 2 and 5 mg/mL following s.c. administration at the dosing of 4 mg/kg (mean ± SD, *n* = 3).

**Figure:**
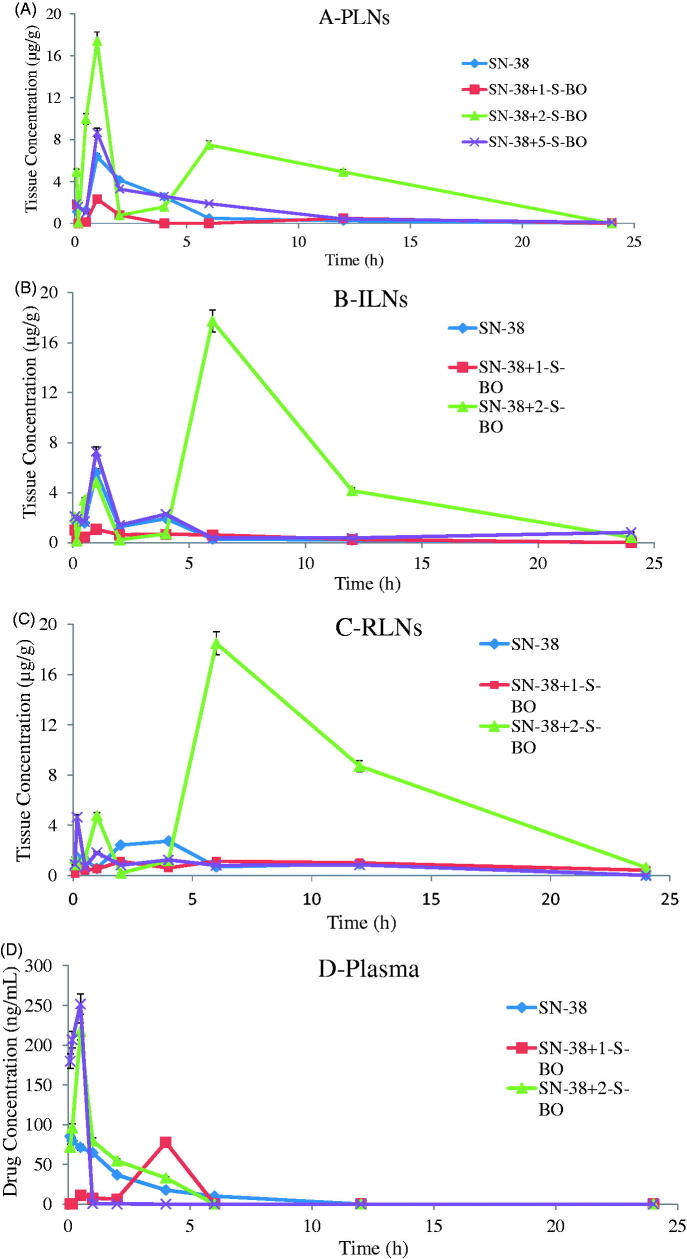

**Figure:**
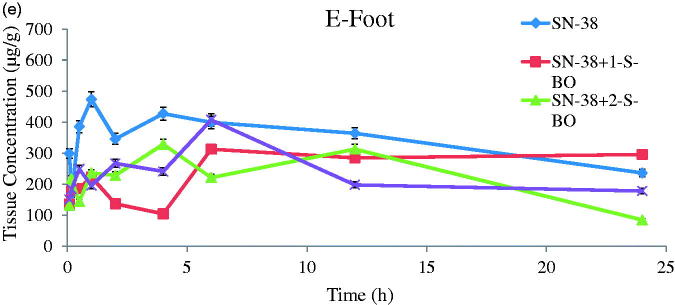

**Table 1.** Non-compartmental pharmacokinetic parameters of injection site, plasma, popliteal lymph nodes (PLNs), iliac lymph nodes (ILNs) and renal lymph nodes (RLNs) in the SN-38-Lips group and SN-38-Lips group combined with S-BO in the concentrations of 1, 2 and 5 mg/mL following s.c. administration at the dosing of 4 mg/kg (mean ± SD, *n* = 6).

		SN-38-Lips	SN-38-Lips+	SN-38-Lips+	SN-38-Lips+
			1-S-BO	2-S-BO	5-S-BO
		Injection site			
AUC_0–24 h_	mg/kg·h	8235.673	6290.983	5496.435	5657.746
AUC_0–∞_	mg/kg·h	16,269.832	13,223.539	7295.406	10,560.689
MRT_0–24 h_	h	10.659	13.073	9.962	10.547
MRT_0–∞_	h	34.043	31.302	18.592	33.085
*t*_1/2_	h	23.571	16.524	14.524	24.32
CLz/F	L/h/kg	0	0	0	0
T_max_	h	1	6	4	6
C_max_	mg/kg	473.299	312.632	328.568	407.063
		Plasma			
AUC_0–24 h_	μg/kg·h	201.643	97.848	290.345	163.285
AUC_0–∞_	μg/kg·h	228.936	159.59	406.256	163.31
MRT_0–24 h_	h	1.866	3.462	11.387	0.375
MRT_0–∞_	h	2.685	3.958	12.104	0.375
*t*_1/2_	h	1.898	0.516	2.436	0.172
CLz/F	L/h/kg	17.472	25.064	9.846	24.493
T_max_	h	0.083	4	0.5	0.5
C_max_	μg/kg	85.093	77.891	217.385	251.644
		PLNs			
AUC_0–24 h_	mg/kg·h	19.619	8.75	66.703	29.632
AUC_0–∞_	mg/kg·h	20.412	14.127	96.423	30.273
MRT_0–24 h_	h	2.964	4.562	5.803	4.887
MRT_0–∞_	h	3.448	5.513	9.504	5.423
*t*_1/2_	h	2.323	0.355	4.027	4.298
CLz/F	L/h/kg	0.196	0.210	0.041	0.132
T_max_	h	1	1	1	1
C_max_	mg/kg	6.369	2.326	17.413	8.661
		ILNs			
AUC_0–24 h_	mg/kg*h	12.818	6.662	117.857	23.099
AUC_0–∞_	mg/kg*h	13.498	8.633	119.797	39.276
MRT_0–24 h_	h	2.982	4.833	8.023	8.428
MRT_0–∞_	h	3.630	5.256	8.361	24.511
*t*_1/2_	h	2.345	5.422	3.365	14.5
CLz/F	L/h/kg	0.296	0.463	0.033	0.102
T_max_	h	1	1	6	1
C_max_	mg/kg	5.612	1.067	17.726	7.308
		RLNs			
AUC_0–24 h_	mg/kg*h	15.773	19.577	163.102	12.049
AUC_0–∞_	mg/kg*h	35.653	28.307	166.637	13.963
MRT_0–24 h_	h	15.006	10.373	9.125	5.233
MRT_0–∞_	h	21.116	20.955	9.551	7.002
*t*_1/2_	h	15.176	14.334	3.602	4.253
CLz/F	L/h/kg	0.112	0.141	0.024	0.286
T_max_	h	4	6	6	0.167
C_max_	mg/kg	2.722	1.134	18.485	4.631

The following parameters were included AUC: area under the plasma–time curve; MRT: the mean residence time; *t*_1/2_: the half-life; V: the distribution volumes of the compartment; CL: clearance; C_max_: maximum plasma concentration; T_max_: time to reach the maximum plasma concentration.

#### 3.5.1. Injection-site retention

As shown in Figure 4(A) and Table 1, SN-38-Lips after s.c. administration mainly remained in the injection site. SN-38 concentration–time curve of the injection site appeared peak value. It indicated there was a process of SN-38-Lips from s.c. injection site into foot pads. SN-38-Lips group firstly absorbed into foot pads reach T_max_ at 1 h with C_max_ of 473.299 mg/kg. The peak concentration levels of 1 and 5 mg/mL S-BO group were both reached immediately at 6 h, with C_max_ of 312.632 and 407.063 mg/kg, respectively. While T_max_ value of 2 mg/mL S-BO group decrease to 4 h in the C_max_ of 328.568 mg/kg. AUC of SN-38 concentration–time curve of the injection site SN-38-Lips group with combination of S-BO in the concentrations of 1, 2 and 5 mg/mL was higher than that in the SN-38-Lips group was and following s.c. administration at the dosing of 4 mg/kg [mean ± standard deviation (SD), *n* = 3]. Further, AUC_0–24 h_ of 2 mg/mL S-BO group (5496.435 mg/kg·h) in the injection sites was lower than 1 mg/mL S-BO group and 1 and 5 mg/mL S-BO group (6290.983 and 5657.746 mg/kg·h). It showed that the ability of SN-38-Lips draining by lymph for lymphatic exposure was improving by 2 mg/mL S-BO. Lower MRT_0–24 h_, *t*_1/2_ and CL values in 2 mg/mL S-BO group showed SN-38-Lips had shorter retention time and faster clearance and elimination in the injection site, implying faster drainage from injection compared to other formulation group. Thus, the drainage from injection site was in descending order of SN-38-Lips with 2 mg/mL S-BO group, SN-38-Lips with 5 mg/mL S-BO group, SN-38-Lips with 1 mg/mL S-BO group and SN-38-Lips group.

#### 3.5.2. Plasma concentration

According to the data got from HPLC analysis shown in Figure 4(A) and Table 1, SN-38 could be detected in plasma after s.c. administration of SN-38-Lips. After S-BO group administration, no drug was detected in plasma after 4 h. However, for SN-Lips group, drug levels in plasma always could be examined during 6 h. All the administration groups could be quickly detected in plasma in 30 min after s.c. administration, respectively. But T_max_ was further increased to 4 h in the 1 mg/mL S-BO group compared to 5 min in SN-38-Lips group with 85.093 μg/kg of C_max_ and 30 min in 2 and 5 mg/mL S-BO group with 217.385 and 251.644 μg/kg of C_max_. After 6 h post-injection, no drug was detected in plasma. It implied that only a small amount of free SN-38 unloaded in the SN-38-Lips could penetrate blood capillaries into systematic circulation. AUC_0–24 h_ of 2 mg/mL S-BO group were 290.345 μg/kg·h which were significantly higher (*p*<.05) than SN-38-Lips group (1.44-fold), 1 mg/mL S-BO group (2.97-fold) and 5 mg/mL S-BO group (1.78- fold), showing that 2 mg/mL S-BO group enhanced systematic exposure of SN-38-Lips. And 2 mg/mL S-BO significantly (*p*<.05) prolonged plasma exposure of SN-38-Lips after s.c. administration, MRT_0–24 h_ and *t*_1/2_ of which were increased to 11.387 and 2.436 h and CL of which were decreased to 9.846 L/h/kg.

SN-38-Lips in the subcutaneous interstitium by s.c. injection had three distribution pathway: the first one is the injection-site retention, the second pathway is the blood capillary drainage and the last one is the lymphatic capillary drainage. The substances injection must traverse the interstitium which was one obstacle of the intra-lymphatic drug delivery system administered interstitially and the gap of lymphatic capillaries was the other barrier (Casley-Smith, 1980). It was a passive process that liposomes transferred from interstitial space into the lymphatic, occurring naturally as the lymph was formed (Cox, 1981). Liposome as lymphatic-targeting drug carrier which had good lymphatic drainage should traverse from the interstitial space and gaps of lymphatic capillaries into lymphatic system (Hawley et al., 1995; Singh et al., 2014) and entered into the systematic circulation as few as possible. Exactly, 2 mg/mL S-BO decreased AUC of SN-38-Lips in the injection site from 8235.673 to 5496.435 mg/kg·h, but only increased AUC in the plasma to 290.345 μg/kg·h. It indirectly showed that large amounts of SN-38-Lips were drained into lymphatic system, verifying the promotion ability of 2 mg/mL S-BO for the lymphatic drainage of SN-38-Lips.

### 3.6. Lymph node uptake and non-compartmental pharmacokinetic analysis

SN-38 concentration–time curve of lymph nodes taken from s.c. injected SN-38-Lips group and SN-38-Lips group combined with 1, 2 and 5 mg/mL of S-BO were shown in Figure 4(C) (PLNs), Figure 4(D) (iliac lymph node, ILNs) and Figure 4(E) (renal lymph node, RLNs), and related non-compartmental pharmacokinetic parameters were summarized in Table 1.

#### 3.6.1. Popliteal lymph node

Lymph nodes distributions of SN-38-Lips measured in PLNs were shown in Figure 4(C). And the non-compartmental pharmacokinetic parameters are shown in Table 1. As shown in Figure 4(C), the double peaks appeared in all SN-38 concentration–time curves of lymph nodes, which were observed in the previous lymphatic target study due to one-way lymphatic circulation (Ye et al., 2014, 2015). The reasonable inference was conducted that drugs in the lymph could finally return to the lymphatic system again through the systemic circulation due to one-way lymphatic circulation. As T_max_ of all administration groups reaching to 1 h, C_max_ of 2 mg/mL S-BO group was higher than other administration. Simultaneously, AUC_0–24 h_ of 2 mg/mL S-BO group were significantly increased (*p*<.05) compared to SN-38-Lips group, 1 mg/mL of S-BO group and 5 mg/mL of S-BO group in the PLNs (3.399-, 7.623-, and 2.251-fold increase). The other important pharmacokinetic parameters in the PLNs, such as *t*_1/2_ and MRT_0–24 h_ were prolonged and CL was slowed significantly (*p*<.05) by S-BO after s.c. administration. After 1 h post-dosing, SN-38 concentrations of SN-38-Lips group in PLNs decreased rapidly; 12 h after administration, the concentration of 2 mg/mL S-BO group was about 21-fold higher than that of SN-38-Lips group. The above results in 2 mg/mL S-BO group showed excellent lymph node uptake and retention in the PLNs, implying that 2 mg/mL S-BO contributed to facilitate SN-38-Lips absorbing into and residing in lymph nodes.

#### 3.6.2. Iliac lymph node

The curves of concentration versus time in ILNs are shown in Figure 4(D). The peak concentrations of SN-38-Lips combined with 1 and 5 mg/mL S-BO reached at 1 h, while T_max_ of 2 mg/mL S-BO group was prolonged to 6 h. C_max_ of 2 mg/mL S-BO group was higher than those of SN-38-Lips group, 1 mg/mL S-BO group and 5 mg/mL S-BO group (9.195-, 17.691-, 5.102- increase), resulting in the largest AUC_0–12 h_ of SN-38 concentration–time curve in the ILNs. It showed that good lymphatic uptake of SN-38-Lips improved via 2 mg/mL S-BO compared with other formulations. For ILNs, MRT_0–24 h_ and *t*_1/2_ of four formulations groups was in descending order of 5, 2, 1 mg/mL S-BO group and SN-38-Lips group, however, the lowest CL appeared in the 2 mg/mL S-BO group, implying slowest clearance of SN-38-Lips in the ILNs. It’s worth noting that AUC_0–12 h_ of 2 mg/mL S-BO group was 117.857 mg/kg·h in the ILNs which is 1.767 times more than that in the PLNs, while C_max_ was similar to that in the PLNs.

#### 3.6.3. Renal lymph nodes

Profiles in RLNs (Figure 4(E)) were similar to those in the ILNs. Figure 4(E) appears double peak phenomenon in the 2 mg/mL S-BO group which also had T_max_ reaching to 6 h in the both ILNs and RLNs_._ The non-compartmental pharmacokinetic parameters in Table 1 showed C_max_ and AUC_0–24 h_ in the RLNs were slightly higher than those in the ILNs (1.043- and 1.384-times, respectively), which still were the most in the four administration groups. Thus, there was the same good level of lymph node uptake for SN-38-Lips in the RLNs compared to in the ILNs by promotion of 2 mg/mL S-BO group.

After entering into the lymphatic system from the injection site, the liposomes were transported by the lymphatic and then were blocked by macrophages phagocytosis and filtration effect in lymph nodes. Thus less retention in injection site and more drug concentration in lymph nodes showed that 2 mg/mL S-BO had good lymphatic drainage and lymph node uptake leading to increasing lymphatic exposure. In the *in vivo* lymphatic exposure study and non-compartmental pharmacokinetic analysis, SN-38-Lips with combination of 2 mg/mL S-BO was found to be superior to other three formulations, speculating which might result from the penetration enhancement of borneol. The borneol could assist other components through the physiological barrier to the targeting tissues or organs, such as through the skin, mucous membranes, gastrointestinal tract and blood–brain barrier by the mechanism of opening the barrier gap (Duan et al., 2016; Zhang et al., 2017). As is known that the gap between blood capillaries was 10–20 nm, and the lymphatic wall was made of monolayer endothelial cells, imbricated arranged and loosely connected, and the gap was 100 nm, which could reach 5 mm when pathological changes appeared. Then, free SN-38 unloaded in the liposome was readily absorbed into draining blood and liposomal SN-38 streamed along with the lymph flow to systematic blood circulation.

### 3.7. Injection site and lymph node irritation

**Figure 5.**
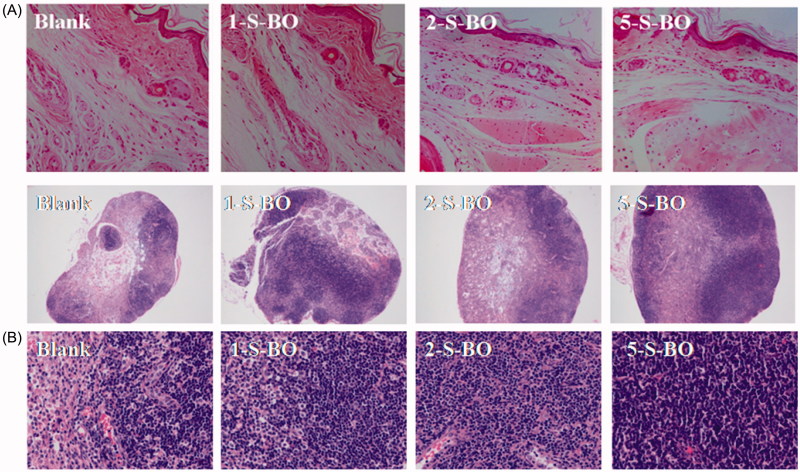
Pathological microphotography of (A) right rear foot pad at ×200K magnification and (B) lymph node at ×200K and ×400K magnification following s.c. administration with S-BO in the concentrations of 1, 2 and 5 mg/mL.

Impact of S-BO on injection site and lymph node irritation was histopathologically investigated via H&E staining. Blank feet and lymph nodes were served as negative controls without administration. The injection site results of histopathological examination in the three borneol group in the concentrations of 1, 2 and 5 mg/mL were shown in Figure 5(A). The lymphatic wall and vein wall were complete and didn’t have any significant expansion. The endothelial cells without swelling and necrosis were clear. There is no obvious inflammation around the lymphatic capillary and capillaries. Muscle structures were normal and the texture was clear. There was no bleeding and edema phenomenon in the interstitial. Thus morphological observation showed three borneol groups in the concentrations of 1, 2 and 5 mg/mL didn’t have obvious irritation for the interstitial tissue. As shown in Figure 5(B), there was no necrosis occurred in the lymph nodes of three administration groups. However, comparing with blank lymph nodes, threes S-BO groups had relative hyperplasia. The number of lymphoid follicles increased, and the germinal center was significantly enlarged and proliferated. There were a large number of various transformed lymphocytes with large nuclei. Therefore, it can be discriminated that there was a reactive hyperplasia in the lymph nodes of the administration group. And the degree of hyperplasia increased with the increase of S-BO concentration. The lymph nodes in the 1 and 2 mg/mL S-BO group only had slight hyperplasia. But the lymph nodes in the 5 mg/mL S-BO group appeared severe hyperplasia, which may be that 5 mg/mL S-BO didn’t not further increase lymph node uptake compared with 2 mg/mL S-BO. Thus, irritability of 2 mg/mL S-BO for lymph nodes could be acceptable.

## 4. Conclusion

In this study, the particle size and zeta potential of SN-38-Lips didn’t showed apparent change when were incubated with S-BO suspension during 12 h, while B-SO could decrease the EE of SN-38-Lips with time, which may due to the fact that S-BO could accelerate the movement of biofilm. The cellular uptake studies showed that 2 and 5 mg/mL S-BO could enhanced fluorescence signal of SN-38-Lips in the cytoplasm of macrophages based on phagocytosis effect in the form of liposomes, especially increased cytoplasmic fluorescence intensity of macrophages in the paracortex zone and medulla of PLN.

The pharmacokinetics parameters of SN-38-Lips in combine with 2 mg/mL S-BO in the lymph node showed excellent lymphatic uptake and retention in the PLNs, ILNs and RLNs, facilitating SN-38-Lips absorbing into and residing in lymph nodes. Less retention in injection site and more drug concentration in lymph nodes also showed that SN-38-Lips combined with 2 mg/mL S-BO had lymphatic drainage effect. Thus, the impact study of S-BO on lymph node uptake of SN-38-Lips *in vitro* and *in vivo* will provide helpful guidance for further study of lymphatic targeting of drug delivery system.

## References

[CIT0001] Ahsan F, Rivas IP, Khan MA, et al. (2002). Targeting to macrophages: role of physicochemical properties of particulate carriers-liposomes and microspheres-on the phagocytosis by macrophages. J Control Release 79:29–40. 11853916 10.1016/s0168-3659(01)00549-1

[CIT0002] Allen TM, Hansen CB, Guo LS (1993). Subcutaneous administration of liposomes: a comparison with the intravenous and intraperitoneal routes of injection. Biochim Biophys Acta 1150:9–16. 8334142 10.1016/0005-2736(93)90115-g

[CIT0003] Casley-Smith JR (1980). The fine structure and functioning of tissue channels and lymphatics. Lymphology 13:177. 7010001

[CIT0004] Cox PH (1981). The kinetics of macromolecule transport in lymph and colloid accumulation in lyirphnodes. Prog Neuropsychopharmacol Biol Psychiatry 2:267–92.

[CIT0005] Cohen MS, Cai S, Xie Y, Forrest ML (2009). A novel intralymphatic nanocarrier delivery system for cisplatin therapy in breast cancer with improved tumor efficacy and lower systemic toxicity *in vivo*. Am J Surg 198:781–6. 19969129 10.1016/j.amjsurg.2009.07.032 PMC2791715

[CIT0006] Cai S, Yang Q, Bagby TR, Forrest ML (2011). Lymphatic drug delivery using engineered liposomes and solid lipid nanoparticles. Adv Drug Deliv Rev 63:901. 21712055 10.1016/j.addr.2011.05.017 PMC3164476

[CIT0007] Chen Z, Gong X, Yang L, et al. (2014). Enhancing effect of borneol and muscone on geniposide transport across the human nasal epithelial cell monolayer. Plos One 9:e101414. 24992195 10.1371/journal.pone.0101414 PMC4081582

[CIT0008] Duan M, Xing Y, Guo J, et al. (2016). Borneol increases blood-tumour barrier permeability by regulating the expression levels of tight junction-associated proteins. Pharm Biol 54:3009–10. 27431008 10.1080/13880209.2016.1199044

[CIT0009] Hawley AE, Davis SS, Illum L (1995). Targeting of colloids to lymph nodes: influence of lymphatic physiology and colloidal characteristics. Adv Drug Deliv Rev 17:129–48.

[CIT0010] Lu Y, Du S, Bai J, et al. (2012). Bioavailability and brain-targeting of geniposide in gardenia-borneol co-compound by different administration routes in mice. Int J Mol Sci 13:14127. 23203054 10.3390/ijms131114127 PMC3509570

[CIT0011] Mai LM, Lin CY, Chen CY, Tsai YC (2003). Synergistic effect of bismuth subgallate and borneol, the major components of Sulbogin, on the healing of skin wound. Biomaterials 24:3005–12. 12895572 10.1016/s0142-9612(03)00126-1

[CIT0012] Moghimi SM, Moghimi M (2008). Enhanced lymph node retention of subcutaneously injected IgG1-PEG2000-liposomes through pentameric IgM antibody-mediated vesicular aggregation. Biochim Biophys Acta 1778:51–5. 17936719 10.1016/j.bbamem.2007.08.033

[CIT0013] Mcallaster JD, Cohen MS (2011). Role of the lymphatics in cancer metastasis and chemotherapy applications. Adv Drug Deliv Rev 63:867–75. 21699937 10.1016/j.addr.2011.05.014

[CIT0014] Oussoren C, Zuidema J, Crommelin DJA, Storm G (1997). Lymphatic uptake and biodistribution of liposomes after subcutaneous injection. ii. Influence of liposomal size, lipid composition and lipid dose. Biochim Biophys Acta 1328:261–72. 9315622 10.1016/s0005-2736(97)00122-3

[CIT0015] Ru G, Han L, Qing J, et al. (2016). Effects of borneol on the pharmacokinetics of 9-nitrocamptothecin encapsulated in PLGA nanoparticles with different size via oral administration. Drug Deliv 23:3417. 27174642 10.1080/10717544.2016.1189466

[CIT0016] Swartz MA (2001). The physiology of the lymphatic system. Adv Drug Deliv Rev 50:3–20. 11489331 10.1016/s0169-409x(01)00150-8

[CIT0017] Singh I, Swami R, Khan W, Sistla R (2014). Lymphatic system: a prospective area for advanced targeting of particulate drug carriers. Expert Opin Drug Deliv 11:211–29. 24350774 10.1517/17425247.2014.866088

[CIT0018] Su J, Lai H, Chen J, et al. (2013). Natural borneol, a monoterpenoid compound, potentiates selenocystine-induced apoptosis in human hepatocellular carcinoma cells by enhancement of cellular uptake and activation of ROS-mediated DNA damage. Plos One 8:e63502. 23700426 10.1371/journal.pone.0063502 PMC3658975

[CIT0019] Tao Y, Tang D, Fan W, et al. (2017). Enhancing both oral bioavailability and brain penetration of puerarin using borneol in combination with preparation technologies. Drug Deliv 24:422. 28165806 10.1080/10717544.2016.1259372 PMC8241152

[CIT0020] Wang S, Ye T, Yang BS, et al. (2013). 7-Ethyl-10-hydroxycamptothecin proliposomes with a novel preparation method: optimized formulation, characterization and *in vivo* evaluation. Drug Dev Industr Pharm 39:393. 10.3109/03639045.2012.683441 22583043

[CIT0021] Willis L, Hayes D, Mansour HM (2012). Therapeutic liposomal dry powder inhalation aerosols for targeted lung delivery. Lung 190:251–62. 22274758 10.1007/s00408-011-9360-x

[CIT0022] Ye T, Zhang WJ, Sun MS, et al. (2014). Study on intralymphatic-targeted hyaluronic acid-modified nanoliposome: influence of formulation factors on the lymphatic targeting. Int J Pharm 471:245–57. 24858386 10.1016/j.ijpharm.2014.05.027

[CIT0023] Ye T, Jiang X, Li J, R YG, et al. (2015). Low molecular weight heparin mediating targeting of lymph node metastasis based on nanoliposome and enzyme–substrate interaction. Carbohydr Polym 122:26–38. 25817639 10.1016/j.carbpol.2014.12.061

[CIT0024] Yang R, Mao Y, Ye T, et al. (2015). Study on enhanced lymphatic exposure of polyamidoamin-alkali blue dendrimer for paclitaxel delivery and influence of the osmotic pressure on the lymphatic targeting. Drug Deliv 23:1. 10.3109/10717544.2015.1041577 26017243

[CIT0025] Yi QF, Yan J, Tang SY, et al. (2016). Effect of borneol on the transdermal permeation of drugs with differing lipophilicity and molecular organization of stratum corneum lipids. Drug Dev Industr Pharm 42:1086. 10.3109/03639045.2015.1107095 26635061

[CIT0027] Yin Y, Cao L, Ge H, et al. (2017). L-Borneol induces transient opening of the blood-brain barrier and enhances the therapeutic effect of cisplatin. Neuroreport 28:506. 28471848 10.1097/WNR.0000000000000792

[CIT0028] Yu XY, Neng PQ, Peng CZ (2007). The enhancing effect of synthetical borneol on the absorption of tetramethylpyrazine phosphate in mouse. Int J Pharm 337:74–9. 17275227 10.1016/j.ijpharm.2006.12.034

[CIT0029] Zhou Y, Li W, Chen L, et al. (2010). Enhancement of intestinal absorption of akebia saponin D by borneol and probenecid *in situ* and *in vitro*. Environ Toxicol Pharmacol 29:229–34. 21787607 10.1016/j.etap.2010.01.004

[CIT0030] Zhang Q, Wu D, Wu J, et al. (2015). Improved blood-brain barrier distribution: effect of borneol on the brain pharmacokinetics of kaempferol in rats by *in vivo* microdialysis sampling. J Ethnopharmacol 162:270. 25582491 10.1016/j.jep.2015.01.003

[CIT0031] Zhang QL, Fu BM, Zhang ZJ (2017). a novel agent that improves central nervous system drug delivery by enhancing blood–brain barrier permeability. Drug Deliv 24:1037. 28687052 10.1080/10717544.2017.1346002 PMC8241164

